# Genetic regulation of gene expression of MIF family members in lung tissue

**DOI:** 10.1038/s41598-020-74121-w

**Published:** 2020-10-12

**Authors:** Laura Florez-Sampedro, Corry-Anke Brandsma, Maaike de Vries, Wim Timens, Rene Bults, Cornelis J. Vermeulen, Maarten van den Berge, Ma’en Obeidat, Philippe Joubert, David C. Nickle, Gerrit J. Poelarends, Alen Faiz, Barbro N. Melgert

**Affiliations:** 1 Department of Chemical and Pharmaceutical Biology, Groningen Research Institute for Pharmacy (GRIP), University of Groningen, Antonius Deusinglaan 1, 9713 AV Groningen, The Netherlands; 2 Department of Molecular Pharmacology, Groningen Research Institute of Pharmacy (GRIP), University of Groningen, Groningen, The Netherlands; 3 Groningen Research Institute for Asthma and COPD (GRIAC), University of Groningen, University Medical Center Groningen, Groningen, The Netherlands; 4 Department of Pathology and Medical Biology, University of Groningen, University Medical Center Groningen, Groningen, The Netherlands; 5 Department of Epidemiology, University of Groningen, University Medical Center Groningen, Groningen, The Netherlands; 6 Department of Pulmonary Disease, University of Groningen, University Medical Center Groningen, Groningen, The Netherlands; 7 Center for Heart Lung Innovation, St. Paul’s Hospital, University of British Columbia, Vancouver, Canada; 8 Quebec Heart and Lung Institute, University of Laval, Quebec, Canada; 9 Department of Global Health, University of Washington, Seattle, WA USA; 10 Gossamer Bio, San Diego, CA USA; 11 Respiratory Bioinformatics and Molecular Biology (RBMB), School of Life Sciences, University of Technology Sydney, Building 4, Room 04.07.418, Thomas St, Ultimo, Sydney, NSW 2007 Australia

**Keywords:** Chronic obstructive pulmonary disease, Functional genomics

## Abstract

Macrophage migration inhibitory factor (MIF) is a cytokine found to be associated with chronic obstructive pulmonary disease (COPD). However, there is no consensus on how MIF levels differ in COPD compared to control conditions and there are no reports on MIF expression in lung tissue. Here we studied gene expression of members of the MIF family *MIF*, D-Dopachrome Tautomerase (*DDT*) and DDT-like (*DDTL*) in a lung tissue dataset with 1087 subjects and identified single nucleotide polymorphisms (SNPs) regulating their gene expression. We found higher *MIF* and *DDT* expression in COPD patients compared to non-COPD subjects and found 71 SNPs significantly influencing gene expression of *MIF* and *DDTL*. Furthermore, the platform used to measure *MIF* (microarray or RNAseq) was found to influence the splice variants detected and subsequently the direction of the SNP effects on *MIF* expression. Among the SNPs found to regulate *MIF* expression, the major LD block identified was linked to rs5844572, a SNP previously found to be associated with lower diffusion capacity in COPD. This suggests that MIF may be contributing to the pathogenesis of COPD, as SNPs that influence *MIF* expression are also associated with symptoms of COPD. Our study shows that *MIF* levels are affected not only by disease but also by genetic diversity (i.e. SNPs). Since none of our significant eSNPs for *MIF* or *DDTL* have been described in GWAS for COPD or lung function, *MIF* expression in COPD patients is more likely a consequence of disease-related factors rather than a cause of the disease.

## Introduction

Macrophage migration inhibitory factor (MIF) is a protein present in many species, which in humans has been identified as a pleiotropic or proinflammatory cytokine^1^. MIF is expressed by many immune and non-immune cell types and in most tissues in humans^2^. Unlike many other cytokines, MIF is produced and pre-stored in intracellular vesicles for rapid release and its release has been shown to be associated with conditions of stress, toxicity and apoptosis^3,4^. Due to MIF’s early associations with inflammation and to its role in cell damage, it has been extensively studied in a variety of human diseases and has been shown to associate with chronic diseases, with MIF levels differing in comparison to healthy conditions^5–8^.

MIF has also been linked to respiratory diseases, including chronic obstructive pulmonary disease (COPD)^6,9^. Yet, the reported data for MIF in COPD appear to be inconsistent. Higher levels of MIF were shown in serum, sputum, and in macrophages present in bronchoalveolar lavage of COPD patients compared to healthy smokers and (non-smoker) controls^10,11^. However, other studies showed lower levels of MIF in serum of COPD patients (GOLD stages II–IV) compared to controls^12^, and also lower plasma MIF levels in COPD patients compared to healthy smokers^13^. Interestingly, *MIF* expression levels were shown to be influenced by the MIF-794 CATT_5–8_ microsatellite (rs5844572), in which 5, 6, 7, or 8 repeats of the CATT sequence could be found and the 5-CATT repeat (CATT_5_ allele) leads to the lowest level of *MIF* expression under basal or stimulated conditions^14^. Additionally, it was reported that the MIF-794 CATT_5_ allele was associated with a lower diffusion capacity in COPD patients^15^. Therefore, genetic variation may explain some of the differences found for MIF expression in COPD and may also influence disease severity as defined by the level of diffusion capacity.

D-Dopachrome tautomerase (DDT, also known as MIF-2) is another member of the MIF protein superfamily that has been suggested to play similar roles to those of MIF^16^. However, studies linking DDT to COPD are lacking. Furthermore, the human genome also encodes a gene known as DDT-like (*DDTL*), which according to genomic records appears to be primarily present in primates^17^. *DDTL* shows high sequence similarity with DDT (approximately 80%), and is located in close proximity to *DDT* and *MIF*. To date, nothing is known about the biological function of DDTL or its expression in lung tissue.

Due to the association of MIF with numerous chronic inflammatory diseases, the scientific community currently has an interest in generating MIF inhibitors to fight chronic diseases^18^. For COPD however, given the inconsistent reports, it is not clear yet whether inhibiting or mimicking MIF would be beneficial. Therefore, it is important to attain more clarity on the levels and regulation of MIF and other MIF family members in lung tissue in COPD.

Here, we aimed to investigate the gene expression levels of the MIF family members MIF, DDT and DDTL in lung tissue of patients with and without COPD and to assess whether their gene expression is regulated by single nucleotide polymorphisms (SNPs).

## Results

### Gene expression of MIF family members in lung samples

**Figure 1:**
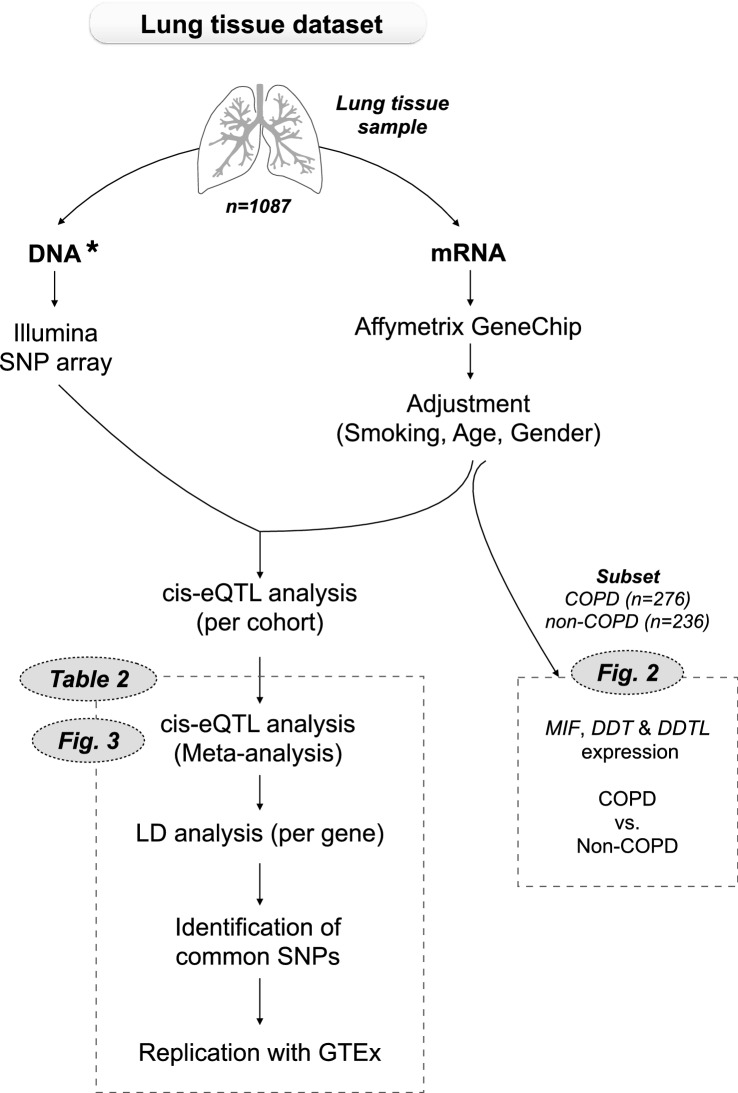
Schematic representation of the lung tissue dataset and methods used in our study. The total lung tissue dataset (n = 1087) was used for the eQTL analysis and a subset of COPD patients (n = 276) and matched non-COPD subjects (n = 236) from the same dataset was used for the gene expression analysis, comparing expression levels of MIF, DDT and DDTL. *DNA was isolated from blood samples in the Laval cohort and from lung tissue samples in the Groningen and British Columbia cohorts.

**Figure 2:**
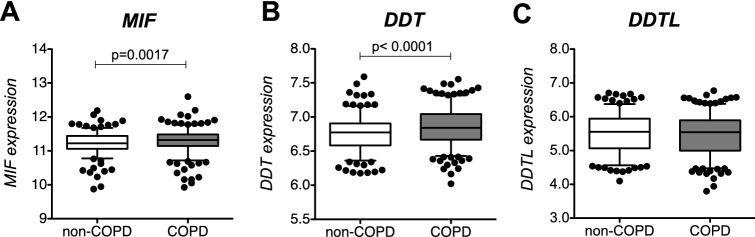
MIF, DDT and DDTL expression in lung tissue from COPD and non-COPD patients. Gene expression profiles for MIF (**A**), DDT (**B**) and DDTL (**C**) were obtained using a custom Affymetrix array (see GEO platform GPL10379), using 276 samples of COPD patients and 236 samples of non-COPD subjects, from the lung tissue database. Units of gene expression (y axis) represent Log2(microarray intensity) units. Data are presented as box and whiskers plots of the 5–95 percentile with median. Statistical differences were tested with Mann Whitney test.

We first compared gene expression levels of *MIF*, *DDT* and *DDTL* in lung tissue samples from a subset of subjects from the lung tissue dataset, with (n = 276) and without COPD (n = 236). An overview of the lung tissue dataset and methods used in our study are shown in Fig. 1. Clinical characteristics of the subjects included for the gene expression analysis are presented in Table 1. Gene expression was adjusted for gender, age and smoking status. We found significantly higher expression of *MIF* (p-value = 0.0017, Fig. 2a) and *DDT* (p-value = 0.0001, Fig. 2b) in subjects with COPD compared to those not having COPD, and no significant differences for *DDTL* expression (Fig. 2c). The higher expression of *MIF* and *DDT* in COPD was driven by patients with COPD GOLD stage 4 for *MIF* and COPD GOLD stage 2 and 4 for *DDT* (Fig. S1). In contrast, we did not find biologically meaningful correlations with either FEV_1_ or FEV_1_/FVC (no r values above 0.1) when either analyzing non-COPD and COPD patients together or separately.

**Table 1:** Characteristics of patients with or without COPD used for gene expression analysis.

	COPD	Non-COPD	p-value
Number	276	236	
Age (years)	64 (56–70)	62 (55–69.75)	NS
Male/female (n)	162/114	132/104	NS
Smokers/ex-smokers	84/192	58/178	NS
Pack-years	41.5 (30–57)	38 (25–49)	0.0007
**GOLD stage (n)**			
I	1	–	
II	197	–	
III	22	–	
IV	46	–	
Not classified	10	–	
FEV_1_ (% predicted)	62.17 (53.15–70.43)	94.31 (87.14–105.6)	0.0001
FEV_1_/FVC (%)	58.33 (50.95–64.06)	75.12 (72.83–78.6)	0.0001

Data are represented as numbers (n) or as median with interquartile range. Differences between groups were tested with Mann–Whitney test for quantitative traits and Chi-square for categorical traits. FEV_1_ and FVC values were obtained before treatment with a bronchodilator.

*NS *not significant.

### eQTL analysis of the lung tissue dataset

**Figure 3:**
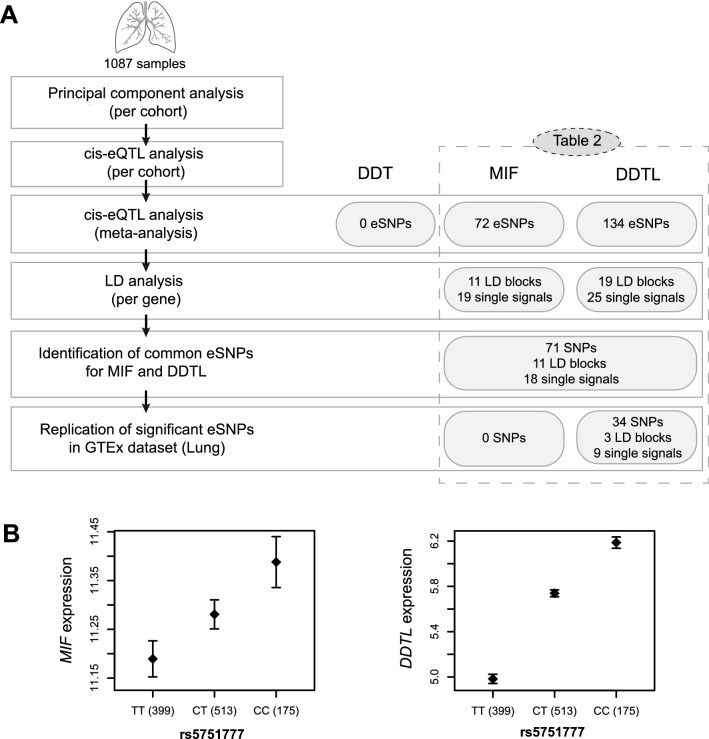
eQTL analysis and main results. (**A**) Schematic representation of the methodology used for the eQTL analysis, subsequent analyses and their corresponding main results. (**B**) eQTL result for rs5751777. Effect of the rs5751777 genotype on MIF and DDTL expression levels in lung tissue samples from the lung tissue dataset (n = 1087). Data are presented as mean ± standard error of the mean.

We then investigated whether *MIF*, *DDT* and *DDTL* expression levels were influenced by the presence of SNPs. To this end, we performed a cis-eQTL analysis^19^ for these three genes using the entire lung tissue dataset (n = 1087), which contains mostly patients with COPD with or without lung cancer, lung cancer patients with normal lung function (Non-COPD controls), and a few patients with a variety of interstitial lung diseases. A schematic representation of the step-by-step approach for the eQTL, subsequent analyses and the main results are shown in Fig. 3a. We only included subjects with both gene expression and genotype data available from the groups described previously^20^.

We found 206 significant SNPs regulating gene expression (eSNPs) in lung tissue, 72 eSNPs for *MIF* and 134 eSNPs for *DDTL*, with p-values as low as 4.09E^−31^ and 1E^−314^, respectively. No significant eSNPs were found for *DDT*. Subsequently, in order to clear the redundant SNPs that are inherited as a cluster, we organized the significant eSNPs in linkage disequilibrium (LD) blocks. We tested this with an LD analysis and found 11 LD blocks and 19 additional independent signals among the 72 eSNPs of *MIF* and 19 LD blocks and 25 independent signals among the 134 eSNPs of *DDTL*. From the SNPs regulating *MIF*, 71 out of 72 were also regulating *DDTL* (11 LD blocks and 18 single signals). All significant SNPs found in our study are shown in Table 2. One of the strongest eSNP blocks for both *MIF* and *DDTL* was represented by rs5751777, which significantly influences expression levels of both *MIF* and *DDTL* as seen in Fig. 3b. We replicated these significant eSNPs using the Genotype-Tissue Expression (GTEx) project^21^, a comprehensive public resource to study tissue-specific gene expression. For this analysis we included only the significant GTEx eQTL data from lung tissue (n = 383). We confirmed 34 of 134 eSNPs for *DDTL* with the same direction of effect (3 LD blocks and 8 independent signals). For *MIF* no eSNPs were confirmed in GTEx with the same direction of effect, whereas we did identify 57 significant *MIF* eSNPs in GTEx with opposite effects (10 LD blocks and 12 independent signals).

**Table 2:** Significant SNPs regulating MIF and DDTL expression in the lung tissue dataset and results for the same SNPs from the GTEx (lung) dataset.

SNP ID	MIF						DDTL					
	Our results			*GTEx*			Our results			*GTEx*		
	FDR	Beta	Genotype of high expression	P value	NES	Genotype of high expression	FDR	Beta	Genotype of high expression	P value	NES	Genotype of high expression
rs140188	4.09E−31	1.05E−01	CC	*3.90E−30*	*7.50E−01*	*GG*	1E−314	6.45E−01	CC	*8.40E−08*	*− 2.60E−01*	*CC*
rs140245	4.72E−31	1.05E−01	AA				1E−314	6.42E−01	AA			
rs113413	1.59E−27	1.12E−01	CC	*4.20E−31*	*− 7.70E−01*	*TT*	1E−314	6.98E−01	CC			
rs6003980	1.01E−22	1.48E−01	AA				3.28E−298	9.18E−01	AA			
rs1006771	2.06E−21	9.27E−02	GG	*4.50E−45*	*8.90E−01*	*TT*	2.04E−270	5.82E−01	GG	*1.90E−08*	*− 2.70E−01*	*GG*
rs5760147	4.68E−18	8.67E−02	CC	*7.70E−35*	*− 8.10E−01*	*AA*	1E−314	6.11E−01	CC			
rs738807	2.93E−17	− 1.20E−01	CC				1.51E−06	− 2.13E−01	CC			
rs140289	1.26E−16	9.29E−02	TT	*1.20E−20*	*7.50E−01*	*CC*	2.98E−104	4.98E−01	TT	*1.20E−06*	*− 2.70E−01*	*TT*
rs5760176	1.50E−16	8.79E−02	GG	*2.20E−36*	*8.40E−01*	*AA*	1.93E−256	6.12E−01	GG	*1.20E−06*	*− 2.40E−01*	*GG*
rs140199	3.99E−15	1.50E−01	TT				1.80E−99	8.15E−01	TT			
rs17004811	1.36E−14	1.51E−01	CC	*8.50E−15*	*− 6.80E−01*	*GG*	1.81E*−*112	8.63E*−*01	CC			
rs1018743	3.40E−08	6.61E−02	GG	*1.50E−14*	*6.50E−01*	*TT*	2.75E−53	3.75E−01	GG			
rs738809	9.10E−05	5.19E−02	GG	*1.50E−09*	*− 4.90E−01*	*AA*	1.53E−51	3.56E−01	GG			
rs915590	7.92E−04	8.48E−02	AA	*1.80E−13*	*7.30E−01*	*GG*	1.36E−39	5.52E−01	AA	*9.50E−09*	*− 3.90E−01*	*AA*
rs1018744	2.61E−03	6.50E−02	TT	*1.40E−11*	*7.50E−01*	*CC*	2.55E−32	4.09E−01	TT	*1.90E−06*	*− 3.60E−01*	*TT*
rs9624364	4.03E−03	8.41E−02	AA				2.88E−20	4.53E−01	AA			
rs2858908	7.69E−03	7.46E−02	AA	*3.10E−06*	*5.00E−01*	*GG*	1.10E−26	4.64E−01	AA	*6.20E−06*	*− 3.30E−01*	*AA*
rs405597	4.59E−02	8.12E−02	CC				3.33E−07	3.22E−01	CC			
rs11703791	3.59E−03	− 1.09E−01	CC				–	–	–			
rs6003909	–	–	–				1.49E−03	1.47E−01	AA			
rs131445	–	–	–				3.29E−03	1.32E−01	CC			
rs9608216	–	–	–				3.70E−03	− 2.40E−01	CC			
rs9620328	–	–	–				8.14E−03	− 1.12E−01	CC			
rs12157360	–	–	–				2.53E−13	2.63E−01	GG			
rs422674	–	–	–				2.47E−06	− 1.47E−01	CC			
rs9608247	–	–	–				3.15E−02	1.24E−01	AA			
**LD blocks**												
rs5751770*	3.64E−22	9.08E−02	TT	*2.50E−55*	*9.20E−01*	*CC*	1E−314	5.96E−01	TT	*4.30E−09*	*− 2.70E−01*	*TT*
rs5751759	2.71E−26	− 1.27E−01	AA				5.05E−12	− 2.40E−01	AA			
rs4461358	2.42E−17	8.34E−02	CC	*8.30E−30*	*− 8.30E−01*	*TT*	4.15E−277	5.81E−01	CC			
rs4822453	1.07E−19	8.72E−02	GG	*2.90E−46*	*8.80E−01*	*TT*	1E−314	5.89E−01	GG	*1.70E−07*	*− 2.50E−01*	*GG*
rs3884794	3.88E−16	8.52E−02	CC	*2.90E−25*	*8.20E−01*	*AA*	4.17E−162	5.37E−01	CC			
rs738806	1.03E−12	7.74E−02	AA	*2.30E−17*	*6.50E−01*	*GG*	1.24E−68	4.05E−01	AA			
rs5760101	1.61E−11	7.53E−02	TT	*2.80E−26*	*7.60E−01*	*CC*	5.27E−113	4.88E−01	TT			
rs2000467	3.94E−10	7.18E−02	AA	*6.40E−46*	*9.00E−01*	*GG*	4.03E−157	5.44E−01	AA	*2.60E−07*	*− 2.50E−01*	*AA*
rs4822461	7.03E−10	8.52E−02	GG	*1.70E−13*	*7.00E−01*	*TT*	1.15E−64	4.81E−01	GG	*1.30E−08*	*− 3.70E−01*	*GG*
rs6004011	2.90E−03	5.56E−02	GG	*4.00E−13*	*− 6.50E−01*	*TT*	9.24E−53	4.39E−01	GG			
rs1984309	1.69E−02	4.18E−02	GG	*3.40E−09*	*5.00E−01*	*AA*	1.31E−36	3.08E−01	GG			
rs5760090	–	–	–				2.89E−58	− 3.84E−01	CC			
rs9612498	–	–	–				6.44E−56	− 3.29E−01	CC			
rs17004046	–	–	–				1.62E−18	2.91E−01	TT			
rs9624472	–	–	–				8.06E−06	2.30E−01	GG			
rs17004049	–	–	–				2.38E−04	2.91E−01	GG			
rs2236624	–	–	–				2.46E−02	− 1.14E−01	CC			
rs5760062	–	–	–				1.13E−02	2.10E−01	CC			
rs9612623	–	–	–				2.05E−02	− 1.02E−01	GG			

Empty spaces indicate that the SNP was not found to be significant in the GTEx dataset. FDR = false discovery rate, eQTL meta-p-value from the lung tissue dataset. Beta = indicates the direction of the eQTL effect per SNP in our study. NES = normalized effect size, indicates the direction of the eQTL effect per SNP from the GTEx dataset. The genotype shown indicates the genotype of each SNP leading to higher MIF or DDTL gene expression. *rs5751770 is a proxy SNP of the LD block rs5751777 (LD coefficient (r^2^) = 0.8477; genotype of high expression in our study for rs5751777: TT; this SNP is not included in the GTEx study).

### MIF splice variants and spliceQTL analysis

**Figure 4:**
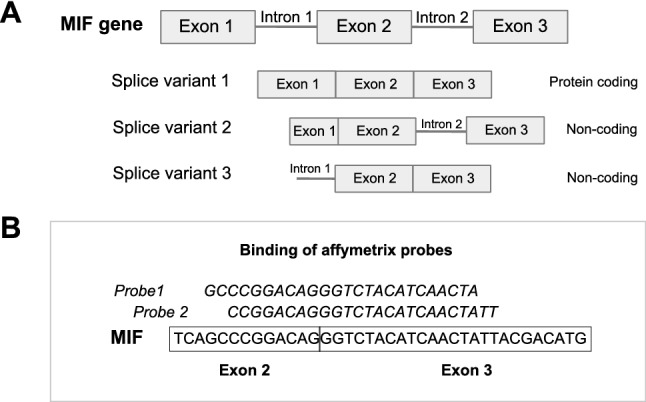
MIF splice variants and binding site of Affymetrix MIF probe. (**A**) Graphic representation of MIF and its splice variants. (**B**) Sequence and binding site of Affymetrix probes for MIF.

A puzzling finding was the difference in effect direction between our study and the GTEx study for the 57 significant *MIF* eSNPs. Considering that our study used microarray and the GTEx study used RNA-Seq to assess gene expression, we investigated the binding site of the microarray *MIF* probe set used in our study*. MIF* has three known splice variants: one protein-coding splice variant which contains 3 exons, and two non-coding splice variants that either retain an intron between exon 1 and 2 or between exon 2 and 3 (Fig. 4a). Interestingly, the *MIF* probe set in our assay targeted the splicing junction Exon2-Exon3 (Fig. 4b), thus detecting only splice variants 1 and 3. Since RNA-Seq would include all splice variant, the opposite eQTL effects may be due to selective expression of *MIF* splice variants.

**Figure 5:**
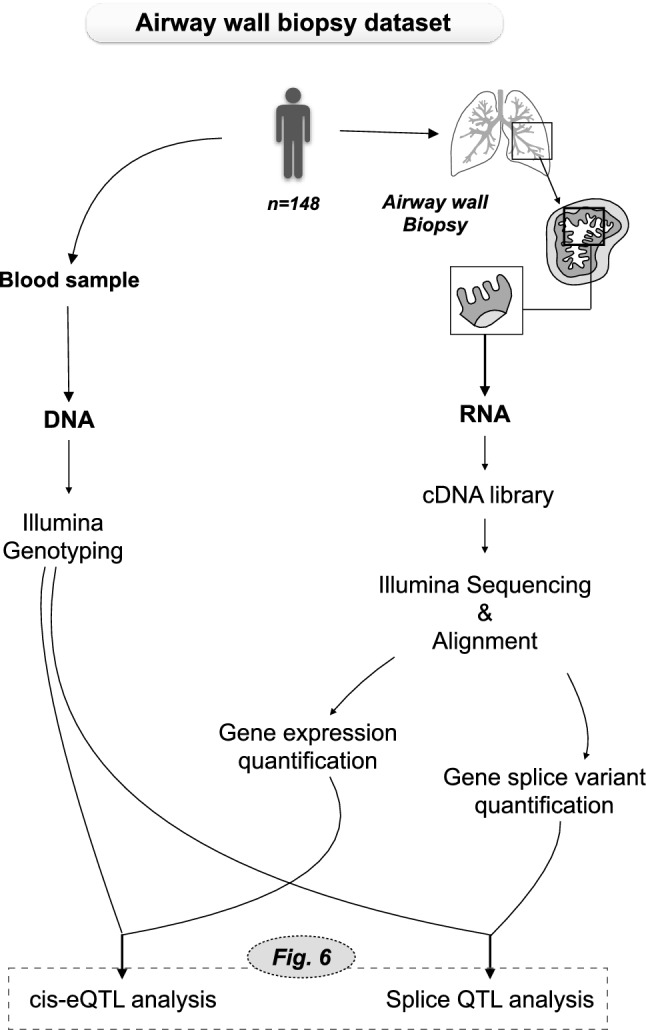
Schematic representation of the airway wall biopsy dataset and methods used in our study. The airway wall biopsy dataset was used for the splice QTL analysis and cis-eQTL analysis in the same dataset. In the current study only results for rs5751777 are shown.

**Figure 6:**
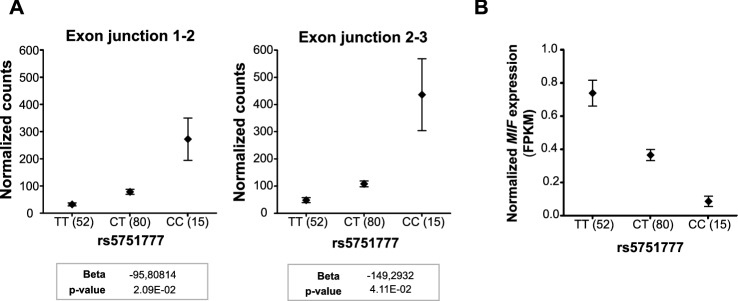
Effect of rs5751777 on expression of MIF splice variants and on total MIF. (**A**) SpliceQTL results. Split read counts mapping across exon–exon junction according to rs5751777 genotype. The number of split reads of a given junction pair was normalized per sample by correcting for variation in library size and transcript abundance in a gene-wise fashion. (**B**) Effect of rs5751777 on normalized MIF expression, represented as fragments per kilobase of exon model per million reads mapped (FPKM). Graphs are presented as mean ± standard error of the mean.

To first confirm the direction of the eQTL effect found in our study with the Exon2-Exon3 junction, we used a previously reported dataset of airway wall biopsies in which the exon–exon reads data were present^22^. Here we evaluated SNP rs5751777, belonging to one of the most significant LD blocks for *MIF,* as a representative of all other *MIF* eSNPs. An overview of the airway wall biopsy dataset and methods used in our study are shown in Fig. 5. We found that in airway wall biopsies, rs5751777 significantly regulated *MIF* split read between Exon 2-Exon 3 with the CC genotype leading to higher expression (Fig. 6a), which is the same direction as in our lung tissue dataset. Additionally, rs5751777 influenced the *MIF* split read between Exon1-Exon 2 in the same direction. Thus indicating that the protein-coding *MIF* splice variant, which contains both exon–exon junctions, is increased by the CC genotype of rs5751777, while the other non-coding splice variants are influenced in the opposite direction. To test this hypothesis we next assessed the eQTL effect of rs5751777 on total *MIF* expression (which includes the protein-coding splice variant and both non-coding splice variants of *MIF*) on the same airway wall biopsies samples. We found indeed a lower expression in the CC genotype for all splice variants together, with the same direction as reported in GTEx (Fig. 6b).

### SNPs in genome-wide association studies (GWAS)

Lastly, we evaluated whether the eSNPs found in our study were reported by the GWAS for COPD or FEV_1_ listed in Table 3. None of our significant eSNPs for *MIF* and *DDTL* (or their LD partners) have been described in GWAS for COPD or lung function.

**Table 3:** List of GWAS on COPD and lung function reported by the GWAS catalog and analyzed in our study.

First author	Year	Study	Disease/trait	References
Pillai, SG	2009	A genome-wide association study in chronic obstructive pulmonary disease (COPD): identification of two major susceptibility Loci	COPD	^26^
Siedlinski, M	2011	Genome-wide association study of smoking behaviours in patients with COPD	Lifetime average and current cigarettes per day, age at smoking initiation, and smoking cessation in COPD	^27^
Cho, MH	2012	A genome-wide association study of COPD identifies a susceptibility locus on chromosome 19q13	COPD	^28^
McDonald, ML	2014	Common genetic variants associated with resting oxygenation in chronic obstructive pulmonary disease	Resting oxygen saturation [SpO_2_] in COPD	^29^
Smolonska, J	2014	Common genes underlying asthma and COPD? Genome-wide analysis on the Dutch hypothesis	COPD; asthma	^30^
Dijkstra, AE	2015	Dissecting the genetics of chronic mucus hypersecretion in smokers with and without COPD	Chronic mucus hypersecretion in heavy smokers with and without COPD	^31^
Hobbs, BD	2017	Genetic loci associated with chronic obstructive pulmonary disease overlap with loci for lung function and pulmonary fibrosis	COPD	^32^
Sakornsakolpat, P	2019	Genetic landscape of chronic obstructive pulmonary disease identifies heterogeneous cell-type and phenotype associations	COPD	^33^
Lutz, SM	2019	Common and rare variants genetic association analysis of cigarettes per day among ever-smokers in chronic obstructive pulmonary disease cases and controls	Average cigarettes per day in COPD	^34^
Repapi, E	2010	Genome-wide association study identifies five loci associated with lung function	Lung function (FEV1 and FEV1/FVC)	^35^
Hancock, DB	2010	Meta-analyses of genome-wide association studies identify multiple loci associated with pulmonary function	Lung function (FEV1 and FEV1/FVC)	^36^
Yao, TC	2014	Genome-wide association study of lung function phenotypes in a founder population	Lung function (FEV1, FVC and FEV1/FVC)	^37^
Liao, SY	2014	Genome-wide association and network analysis of lung function in the Framingham Heart Study	Lung function (FEV1 and FVC)	^38^
Lutz, SM	2015	A genome-wide association study identifies risk loci for spirometric measures among smokers of European and African ancestry	Lung function (PostBD FEV1 and FEV1/FVC ratio)	^39^
Soler Artigas, M	2015	Sixteen new lung function signals identified through 1000 Genomes Project reference panel imputation	Lung function (FEV1, FVC and FEV1/FVC)	^40^
Wain, LV	2015	Novel insights into the genetics of smoking behaviour, lung function, and chronic obstructive pulmonary disease (UK BiLEVE): a genetic association study in UK Biobank	FEV1 and smoking behaviour	^41^
de Jong, K	2015	Genome-wide interaction study of gene-by-occupational exposure and effects on FEV1 levels	FEV1 in occupational exposure	^42^
de Jong, K	2017	Genes and pathways underlying susceptibility to impaired lung function in the context of environmental tobacco smoke exposure	FEV1 in environmental tobacco smoke	^43^
Suh, Y	2017	Genome-wide association study for genetic variants related with maximal voluntary ventilation reveals two novel genomic signals associated with lung function	Lung function (inspiratory muscle strength -maximal voluntary ventilation)	^44^
Wyss, AB	2018	Multiethnic meta-analysis identifies ancestry-specific and cross-ancestry loci for pulmonary function	Lung function (FEV1, FVC and FEV1/FVC)	^45^
Li, X	2018	Genome-wide association study of lung function and clinical implication in heavy smokers	Lung function (PostBD FEV1 and FEV1/FVC ratio)	^46^
Shrine, N	2019	New genetic signals for lung function highlight pathways and chronic obstructive pulmonary disease associations across multiple ancestries	Lung function (FEV1, FVC and FEV1/FVC)	^47^

## Discussion

The primary objective of this study was to evaluate gene expression of the MIF family members *MIF*, *DDT* and *DDTL* in lung tissue of COPD patients compared to non-COPD subjects and to elucidate whether *MIF* expression in lung tissue is regulated genetically by SNPs. We found higher gene expression levels of *MIF* and *DDT* in lung tissue samples of COPD patients, compared to non-COPD subjects. While previous studies have not assessed MIF levels in lung tissue, higher levels of MIF have also been reported in serum, sputum and macrophages of bronchoalveolar lavage of COPD patients^10,11^. However, other studies have also detected lower levels of MIF in serum and plasma of COPD patients compared to controls^12,13^, which could be due to patient selection or the different nature and origin of the samples these studies used. MIF has been described as a proinflammatory cytokine, but there is also evidence that MIF can exert many other functions related to cell survival and anti-apoptosis^12,23,24^. Interestingly, MIF may actually be involved in tissue repair more than in promoting inflammation, but its role varies across lung diseases as discussed in detail in our recent review^9^. In fact, it was previously reported that MIF-deficient mice spontaneously develop age-related emphysema^13^, which suggests that in lung tissue MIF may protect against tissue destruction. The higher *MIF* expression we found in COPD could therefore be a result of activation of protective mechanisms triggered to combat tissue destruction in COPD development. Due to the various shared biological functions between MIF and DDT, and the observed higher *MIF* and *DDT* expression in COPD, it seems likely that DTT may also play a protective role in the lung. However, this is still a hypothesis and requires further testing.

We did not find differences in expression between COPD and non-COPD for DDTL, but our eQTL results suggest that the regulation of *DDTL* expression resembled that of *MIF*. Considering that *MIF* and *DDTL* are located in close proximity (less than 0.1 Mb between the two genes), it is not surprising that our cis-eQTL analysis, which identifies SNPs within 1 Mb from the binding site of the probe, found SNPs regulating the expression of both genes, indicating possible dual regulation. Surprisingly, we did not find significant eSNPs for *DDT*, which is also in the vicinity of *MIF* and *DDTL*. This suggests that there are different regulatory mechanisms between *MIF* and *DDT* and that *DDT* expression is not regulated by the genetic mechanisms included in our study.

To validate our findings, we replicated our eQTL results with the publicly available GTEx dataset. While we confirmed a group of eSNPs for *DDTL*, to our surprise we found a group of significant eSNPs for *MIF* with opposite effects in our study and in the GTEx dataset. The most likely explanation for these opposite effects of *MIF* eSNPs is that different splice variants were present in the quantitation of gene expression in our study and in the GTEx. The MIF probe set in the gene expression array of our lung tissue dataset binds across an exon–exon junction (exon2-exon3; Fig. 4b) and thus can only detect splice variants 1 and 3 and not splice variant 2 (Fig. 4a). The GTEx study assessed gene expression by RNAseq but did not perform a spliceQTL analysis to study the effect of SNPs on each of the splice variants. Focusing on one of the most significant SNP blocks for *MIF* and *DDTL,* rs5751777, and using the airway wall biopsy dataset we were able to assess the effect of this SNP on the expression of all splice variants combined or just variants for each exon–exon junction. We confirmed that the direction of the eQTL effect for the sets of *MIF* splice-variants is opposite to that of all *MIF* splice variants combined and that the effect of the total *MIF* matches the direction of the effect found by GTEx. Even though we could not investigate this splice variant effect in our lung tissue dataset directly, due to the lack of splice variant data, these results do show that the effect of rs5751777 on *MIF* expression has the same direction in the airway wall dataset and in the lung tissue dataset, suggesting the difference with GTEx is indeed cause by splice variants. While this was only tested for rs5751777, this effect also affected all other MIF eSNPs described in Table 2. This highlights the complexity of genetic studies and the importance of measuring *only* protein-coding splice variants for more relevant interpretation of expression data. Nonetheless, it is unknown whether the non-coding splice variants of *MIF* have a different yet relevant molecular function, which may be a point of interest for future studies on MIF.

We found higher *MIF* expression in COPD patients and also found that *MIF* expression can be regulated by SNPs, we therefore investigated whether those SNPs or their LD partners are genetically predisposing individuals for the development of COPD. To that end we evaluated whether any of the significant eSNPs found in our study were reported in GWAS for COPD or lung function, available in the GWAS catalog from the European Bioinformatics Institute. None of our significant eSNPs for *MIF* or *DDTL* have been described in GWAS for COPD or lung function, suggesting that these SNPs are not predominant in COPD patients and most likely do not confer susceptibility for the development of COPD. It is therefore unlikely that the differences in MIF mRNA expression are due to a predominant presence of these SNPs in COPD patients but could be due to a combination of the SNP and environment. We hypothesize that the differential expression in *MIF* and *DDT* between COPD and control patients may be the result of epigenetic regulation, likely caused by multiple factors, but this theory requires further testing.

We also found that the higher *MIF* and *DDT* expression in COPD patients was driven by GOLD stage 4 for *MIF* and stages 2 and 4 for *DDT*, but no correlation with FEV_1_ of FEV_1_/FVC. The fact that MIF knock out mice develop emphysema suggests that the role of MIF may indeed be more related to parenchymal lung tissue than airways^13^. This peripheral role is also suggested by the finding that rs5844572, the MIF-794 CATT_5-8_ microsatellite, was associated with low diffusion capacity and incidentally is in high LD with the LD block rs5751759 we identified for *MIF* and *DDTL*. Unfortunately, no data on diffusion capacity were available in our datasets to confirm said association. Nonetheless, this suggests that the eSNPs located in this LD block not only regulate *MIF* and *DDTL* gene expression but are also linked to a low diffusion capacity, which is associated with disease severity in COPD patients.

In summary, we have shown that COPD patients have higher mRNA expression levels of *MIF* and *DDT* and similar *DDTL* expression in lung tissue, compared to non-COPD subjects. In addition, we have shown that expression of *MIF* and *DDTL* in lung tissue is at least partially controlled genetically and some of these eSNPs are shared between these two genes. This is interesting because little is known about the biological function of DDTL and this provides a basis for understanding the regulation of *DDTL* expression. Moreover, we found that eSNPs for *MIF,* as demonstrated for rs5751777, can have a significant effect on gene expression but that the direction of such effect is influenced by the *MIF* splice variants included in the analysis.

Due to the lack of protein data in our datasets we do not know whether higher MIF and DDT gene expression translates into higher protein levels. Moreover, we did not have epigenetic data in our datasets, which would have allowed us to look into possible epigenetic mechanisms differentially regulating MIF and DDT gene expression between COPD and non-COPD patients. Therefore, there is need for further investigation to identify the main cause and reason for the higher expression of *MIF* and *DDT* in lung tissue of COPD patients. Given the complexity of COPD pathogenesis and the fact that MIF has been shown to be involved in diverse cellular processes, we believe that the high levels of MIF in lung tissue of COPD patients could be the consequence of multiple factors associated with this disease (e.g. injury, oxidative stress, cellular senescence). Based on our current knowledge on various causes and effects of MIF expression, it is currently unclear whether increasing or decreasing MIF expression as a therapeutic strategy would be beneficial for COPD or other chronic lung diseases.

In conclusion, *MIF* expression is not only influenced by the presence of disease (COPD) but also by the eSNPs we identified here. While these SNPs do not appear to be the cause of the gene expression differences observed in our cohort, our data suggest that genetic diversity (i.e. SNPs) could contribute to the discrepancies in the MIF levels reported in COPD studies. Future studies aiming to assess MIF levels and their association with diseases should take into consideration the SNPs reported in our study, as they can have an additional effect on the gene expression levels already influenced by pathological processes. Moreover, it is important to consider that the direction of the effect of SNPs on *MIF* expression is influenced by the *MIF* splice variants detected and care should be taken to distinguish between protein-coding and non-coding variants.

## Materials and methods

### Lung tissue dataset

We used the lung tissue dataset from the Universities of Groningen, Laval and British Columbia^25^. A description of sample collection, demographics of the dataset and gene expression and genotyping analysis has been published previously^19,25^. Briefly, lung tissue was collected from patients with diverse lung diseases who underwent lung resection surgery at Laval University, University of British Columbia, and University of Groningen. All lung tissue samples were obtained in accordance with Institutional Review Board guidelines at the three sites. All patients provided written informed consent and the study was approved by the ethics committees of the Institut universitaire de cardiologie et de pneumologie de Quebec (Laval) and the UBC-Providence Health Care Research Institute Ethics Board (British Columbia). The study protocol was consistent with the Research Code of the University Medical Center Groningen and Dutch national ethical and professional guidelines. RNA was isolated from lung tissue samples and DNA was isolated from the same lung tissue (British Columbia and Groningen) or from blood samples (Laval). RNA converted to fluorescently labeled cDNA was hybridized to a custom Affymetrix HU133 array (see GEO platform GPL10379) and DNA sample was genotyped on the Illumina Human1M-Duo BeadChip array. From this dataset, the samples from 1087 subjects passed all quality controls for DNA and RNA analysis and were included in our study. An overview of this dataset and associated methods is shown in Fig. 1. This dataset has been deposited in the National Center for Biotechnology Information’s Gene Expression Omnibus repository and is accessible through GEO Series accession number GSE23546.

### Airway wall biopsy dataset

A description of sample collection and demographics of this dataset has been published previously^22^. Briefly, bronchial biopsies were taken from segmental divisions of the main bronchi from healthy subjects and asthmatic patients. All protocols were approved by the University Medical Center Groningen medical ethics committee and all subjects provided written informed consent. RNA was isolated from the biopsies using AllPrep DNA/RNA Mini kit (Qiagen) and RNA samples were further processed using the TruSeq Stranded Total RNA Sample Preparation Kit (Illumina). The cDNA fragment libraries were then loaded in pools of multiple samples unto an Illumina HiSeq2500 sequencer. The gene level quantification was performed by HTSeq (version 0.6.1p1) using Ensembl version 75 as gene annotation database. DNA from blood samples was genotyped on Illumina genotyping platforms and submitted to the Michigan imputation server. Imputation was performed using the HRC r1.1 2016 reference panel, the Eagle v2.3 Phasing algorithm, the EUR (European) population parameter and the quality control plus imputation mode. From this dataset, the samples from 148 subjects passed all quality controls for DNA and RNA analysis and were included in our study. An overview of this dataset and associated methods is shown in Fig. 5.

### Gene expression analysis

For the gene expression analysis of the current study, data from the lung tissue dataset was used. Unfiltered gene expression was normalized with the Robust Multichip Average method implemented in the Affymetrix Power Tools softwareV.1.8.5. The Log2(microarray intensity) values of gene expression are used for all subsequent analyses. Gene expression data were adjusted for age, gender and smoking status, using a robust linear model. For gene expression analysis (*MIF*, *DDT* and *DDTL*) a subset of COPD patients and non-COPD control subjects from the lung tissue dataset was selected. COPD was defined as an FEV_1_/ FVC ratio < 70%. Non-COPD was defined as an FEV_1_/FVC ≥ 70% predicted and subjects were selected to match COPD patients as closely as possible on age, gender and smoking status. From both groups, current and ex-smokers of > 40 years with ≥ 5 pack-years were included. For FEV_1_ and FEV_1_/FVC, pre-bronchodilator values were used. Subjects with other lung diseases such as asthma, cystic fibrosis or interstitial lung diseases were excluded. Gene expression values in COPD and non-COPD patients were tested for normal distribution with a Kolmogorov–Smirnov test. These data did not have a normal distribution; therefore differences in gene expression levels between groups were tested with a Mann Whitney *U* test and correlations between gene expression and other parameters were performed with a nonparametric Spearman correlation analysis.

### Expression quantitative trait loci (eQTL) analysis

To identify SNPs significantly regulating gene expression of *MIF*, *DDT* and *DDTL*, a cis-eQTL analysis was performed. Here cis-eQTL is defined as the SNPs significantly associated with gene expression and located within 1 Mb from either side of the binding side of the probe. The eQTL analysis was performed using the lung tissue dataset, as described previously^19^. Briefly, the association between SNPs and the 2-log transformed gene expression of *MIF*, *DDT* and *DDTL* was tested in each cohort (Laval, British Columbia and Groningen). Subsequently, an eQTL for all cohorts was calculated and a Bonferroni-adjusted p-value < 0.05 was used as a significance threshold to correct for multiple testing. An overview of the methods and the step-by-step approach are shown in Figs. 1 and 3a, respectively.

### Linkage disequilibrium analysis

Linkage disequilibrium (LD) between eSNPs for *MIF* and *DDTL* was tested with the LDlink tool from the National Institutes of Health of the United States (https://ldlink.nci.nih.gov), for European populations using an R^2^ threshold of 0.8. The SNPs belonging to the same LD block (R^2^ > 0.8) were clustered together and the remaining SNPs were catalogued as independent signals.

### MIF splice variants and spliceQTL

The effect of eSNPs on *MIF* splice variant expression was performed with the airway wall biopsy dataset. Using the MatrixEQTL package it was determined whether SNP allele dosages were associated with split read counts of splice junction pairs within 1 Mb of the SNP (Cis-SpliceQTL). Predicted dosage of the alternative allele was used as the explanatory variable and age, gender and current smoking status were set as covariables. SNPs with a minor allele frequency lower than 0.05 were removed. A custom script was used to quantify split reads in the sequence alignment map files, as identified by the N-operation in the CIGAR string. Splice junction pairs identified by split reads were grouped by strand, start and end position of the intron and annotated to the host gene. Number of split reads of a given junction pair were normalized per sample by correcting for variation in library size and transcript abundance in a gene-wise fashion.

### Identification of significant eSNPs in GWAS for COPD

To identify the presence of the significant eSNPs from our study in GWAS for COPD, the LD partners of all SNPs found in our study were identified with the LDproxy tool of the National Institutes of Health of the United States (https://ldlink.nci.nih.gov) for European populations. All significant SNPs found in our study and all proxy variants reported by the LDproxy analysis were cross-referenced with the list of SNPs reported in the GWAS catalog from the European Bioinformatics Institute (https://www.ebi.ac.uk/gwas/; reporting SNPs with p-value < 1 × 10^–5^) found by the GWAS for FEV_1_ and COPD listed in Table 3.

## Supplementary information

Supplementary Figure 1.

## Data Availability

The lung tissue dataset analyzed in the current study is available in the National Center for Biotechnology Information’s Gene Expression Omnibus repository and is accessible through GEO Series accession number GSE23546. Sequence data from the bronchial biopsy dataset analyzed in the current study has been deposited at the European Genome-phenome Archive (EGA), which is hosted by the EBI and the CRG, under accession number EGAS00001003735.
