# Supplementary material for: Genetic regulation of gene expression of MIF family members in lung tissue
Source: Sci Rep. 2020 Oct 12;10:16980. doi: 10.1038/s41598-020-74121-w (PMC7552402; doi:10.1038/s41598-020-74121-w)
Supplement: Supplementary file 1 — Supplementary Figure 1. [file 41598_2020_74121_MOESM1_ESM.pdf]

SUPPLEMENTARY INFORMATION

Title:
Genetic regulation of gene expression of MIF family members in lung tissue

Authors:
Laura Florez-Sampedro, Corry-Anke Brandsma, Maaike de Vries, Wim Timens, Rene Bults, Cornelis J. Vermeulen, Maarten van den Berge, Ma'en Obeidat, Philippe Joubert, David C. Nickle, Gerrit J. Poelarends, Alen Faiz, Barbro N. Melgert

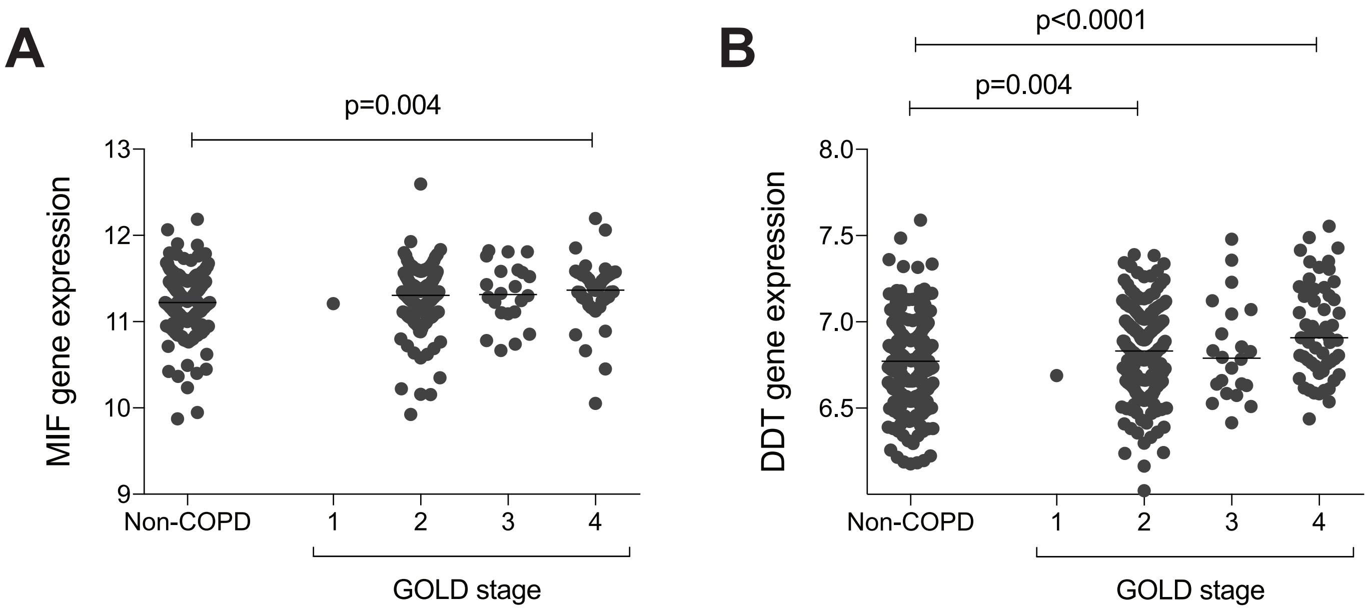

Supplementary figure 1. MIF and DDT gene expression in lung tissue from non-COPD patients and from COPD patients according to GOLD stages. Gene expression profiles for MIF **(A)** and DDT **(B)** were obtained using a custom Affymetrix array (see GEO platform GPL10379), using 276 samples of COPD patients and 236 samples of non-COPD subjects, from the lung tissue dataset. Units of gene expression (y axis) represent Log2(microarray intensity) units. Data are presented as Scatter plot with median. Statistical differences were tested with Kruskal-Wallis and Dunn's test to correct for multiple comparisons. Only significant differences ($p<0.05$) are shown.
